# The microRNA pathway regulates obligatory aestivation in the cabbage stem flea beetle *Psylliodes chrysocephala*

**DOI:** 10.1038/s42003-025-08721-5

**Published:** 2025-08-27

**Authors:** Gözde Güney, Kerstin Schmitt, Johan Zicola, Umut Toprak, Michael Rostás, Stefan Scholten, Doga Cedden

**Affiliations:** 1 Agricultural Entomology, Department of Crop Sciences, University of Göttingen, Göttingen, Germany; 2 Institute of Microbiology and Genetics, Department of Molecular Microbiology and Genetics, University of Göttingen, Göttingen, Germany; 3 Göttingen Center for Molecular Biosciences (GZMB), Service Unit LCMS Protein Analytics, University of Göttingen, Göttingen, Germany; 4 Division of Crop Plant Genetics, Department of Crop Sciences, University of Göttingen, Göttingen, Germany; 5 Faculty of Agriculture, Department of Plant Protection, Ankara University, Ankara, Turkey; 6 Department of Evolutionary Developmental Genetics, Johann-Friedrich-Blumenbach Institute, Göttingen Center for Molecular Biosciences, University of Göttingen, Göttingen, Germany

**Keywords:** Animal physiology, Entomology

## Abstract

Aestivation, or summer diapause, is a dormancy strategy that enables animals to survive hot and dry summer conditions. Despite its ecological importance, the gene regulatory mechanisms underlying aestivation remain poorly understood. MicroRNAs (miRNAs) are post-transcriptional regulators involved in various biological processes, including development. Here, we investigated the role of miRNAs in obligatory aestivation in the cabbage stem flea beetle (*Psylliodes chrysocephala*), a major pest of oilseed rape. Small RNA sequencing revealed that approximately 25% of miRNAs were differentially abundant during aestivation. RNA interference-mediated inhibition of the miRNA pathway, combined with proteomics, identified 116 miRNA-regulated proteins involved in metabolic and catabolic processes. Integrated transcriptome analysis suggested that 71% of these miRNA-regulated proteins were also downregulated at the mRNA level, while the remaining 29% were likely regulated primarily through translational inhibition. Degradome sequencing confirmed miRNA-mediated regulation of several transcripts and suggested exonucleolytic decay as the predominant mechanism. Disruption of the miRNA pathway impaired key aestivation traits, including metabolic suppression, changes in body composition, behavior inhibition, and heat tolerance. These findings suggest a central role for miRNAs in regulating insect dormancy, with implications for understanding climate change impacts on insect physiology and for developing novel pest control strategies.

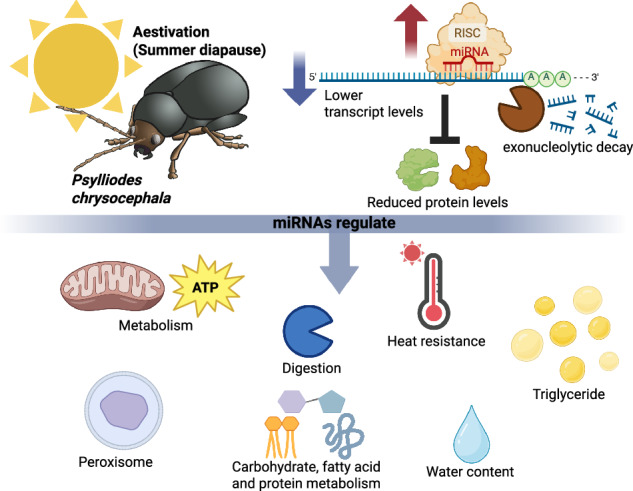

## Abstract (web summary)

A study integrating sRNA-seq, proteomics, degradomics, and RNAi suggests that miRNAs regulate aestivation-related proteins, including those related to metabolism, and are important for heat resistance in the cabbage stem flea beetle.

## Introduction

The cabbage stem flea beetle (CSFB), *Psylliodes chrysocephala* (Coleoptera: Chrysomelidae), is a key pest of winter oilseed rape (*Brassica napus napus*) in Northern Europe^1,2^. CSFB eggs hatch in late autumn, and larvae feed by tunnelling into the petioles and stems of oilseed rape. After overwintering as larvae, they pupate in the soil in late spring and adults emerge in early summer and feed on oilseed rape leaves^3–5^. CSFB adults exhibit obligatory aestivation, also known as summer diapause, meaning that regardless of environmental and rearing conditions, newly emerged adults enter a preparatory (pre-aestivation) phase and, within 15 days, enter aestivation, which lasts from mid to late summer in nature^6^. Aestivating CSFB has dramatically reduced metabolic rate and feeding activity, and instead, it relies on internal energy reserves (e.g. lipid stores) to maintain its suppressed metabolism. During aestivation, CSFB demonstrates resilience to elevated temperatures and reduced humidity, conditions characteristic of European summers^6^. Only post-aestivation CSFB can reproduce, making aestivation a key stage from a pest management perspective. Previously, we identified thousands of differentially abundant transcripts in aestivation compared to non-aestivation stages, which were associated with key aestivation phenotypes, including lower metabolic rate^6^. Yet the mechanisms underlying this gene regulation remained uncharacterised. A mechanism that could be involved in this regulation is the microRNA (miRNA) pathway due to its well-established role in gene regulation.

Most biological phenomena depend on specific gene expression patterns that are regulated by epigenetic mechanisms^7^. The miRNA pathway has been recognised as a key post-transcriptional regulator that enables intricate control of gene expression, complementing canonical epigenetic mechanisms such as histone modifications^8,9^. In animals, the miRNA pathway begins with the transcription of the primary (pri)-miRNA, a 5′-capped and 3′ polyadenylated transcript, by RNA polymerase II in the nucleus. The pri-miRNA is processed by the enzyme Drosha into pre-miRNA hairpins (~100 nt), which are then transported into the cytoplasm via Exportin-5^10,11^. In the cytoplasm, the enzyme Dicer-1 further processes pre-miRNA, releasing the miRNA/miRNA* duplex (~22 nucleotides)^12,13^. One of the strands, known as the mature miRNA, is retained by the Argonaute-1 (Ago-1), forming the RNA-induced silencing complex (RISC)^14^. miRNA-loaded-RISC typically interacts with 3′ untranslated regions (UTR) of mRNA that are recognised mainly by the seed sequence (2–8 nucleotides from the 5′ end) of the miRNA, resulting in decay or translational inhibition of target mRNAs in animals^15^.

The miRNA pathway has been implicated in the regulation of various biological processes in animals, including development and metabolism^16–23^ because the expression of miRNA fluctuates between different life stages^24–27^. Previous studies in insects have also suggested that miRNA could regulate diapause and aestivation^28–31^. miRNA-mediated regulation of diapause is plausible because this dormant state necessitates the downregulation of numerous genes, a process that can be mediated by the expression of specific miRNA. However, in non-model organisms, molecular methods are mostly limited to studying miRNA that are differentially abundant between diapausing and non-diapausing stages through small RNA sequencing^28–30^. Nevertheless, multi-omics approaches, including the integration of transcriptomics and proteomics, have been applied in insects to elucidate the genetic regulation of diapause^32,33^ and could similarly provide deeper insights into its regulation by miRNA.

In this study, we hypothesised that a subset of genes that are required to be suppressed during aestivation are regulated via the miRNA pathway, as this pathway is known to regulate many biological processes through gene suppression. To that end, we first sequenced small RNA from the three adult stages of CSFB and identified differentially abundant miRNA in aestivation. Next, we knocked down the expression of two core miRNA pathway genes via RNA interference (RNAi) and identified deregulated proteins via quantitative proteomics. RNA degradome was sequenced to find evidence for transcript decay mediated by differentially abundant miRNA. Also, the putative targets of the identified miRNA were predicted through in silico target prediction based on sequence complementarity to 3′ UTRs of aestivation-associated transcripts. Finally, we investigated the effects of the inhibition of the miRNA pathway on the aestivation phenotype, including resilience to summer conditions and metabolic alterations.

## Results

### Changes in the abundance of miRNA in aestivation

**Fig. 1:**
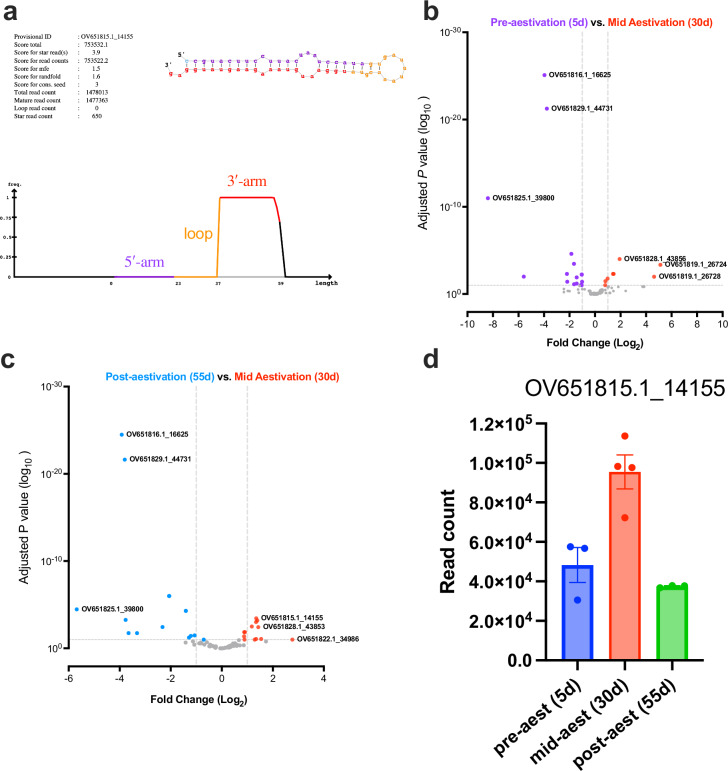
Differentially abundant miRNA at different life stages. **a** An example plot produced using miRdeep2 for the identification of a miRNA which was highly expressed in aestivation compared to other stages. The predicted RNA structure of the pre-miRNA and frequency of read counts corresponding to the mature (red), loop (orange) and passenger (purple) parts of the duplex are shown. DESeq2 analysis was conducted on miRNA identified and quantified using miRdeep2 in pre-aestivation (*n* = 3), aestivation (*n* = 4) and post-aestivation (*n* = 3) cabbage stem flea beetle females. **b** Differentially abundant miRNA in the comparison between aestivation and pre-aestivation. **c** Differentially abundant miRNA in the comparison between aestivation and post-aestivation. **d** Normalised expression count (mean ± SEM) of the example miRNA at three different life stages; pre-aestivation (pre), aestivation (aest) and post-aestivation (post).

CSFB obligatorily enters aestivation within 2 weeks following adult eclosion, dividing adulthood into three stages: pre-aestivation (first 2 weeks), aestivation (15- to 40-day-old adults) and post-aestivation (older than 40-day-old adults). We conducted small RNA-seq in these three stages to de novo identify miRNA and investigate differentially abundant miRNA. In total, we identified 105 mature miRNA in CSFB adults based on mirdeep2 scores (Supplementary Data 1), which take into account mature RNA counts and randfold value for the miRNA precursor (an example is provided in Fig. 1a), and 69 of these showed homology to miRNA identified in red flour beetle *Tribolium castaneum*, in line with the previous analysis that identified 65 conserved miRNA families among insects^34^.

In the pre-aestivation vs. aestivation comparison, we identified 8 and 17 miRNA with significantly higher and lower abundance in aestivation, respectively (adjusted *P* < 0.05 by DeSeq2, Fig. 1b). The post-aestivation vs. aestivation comparison identified 13 and 15 miRNA with significantly higher and lower abundance in aestivation, respectively (adjusted *P* < 0.05 by DeSeq2, Fig. 1c). Four miRNA had significantly higher abundance in aestivation compared to both pre- and post-aestivation stages (an example is provided in Fig. 1d). Interestingly, we observed that the precursors of two miRNA increasing in abundance in aestivation compared to both other stages mapped 24.7 kb apart on chromosome 16 (Accession: OV651828.1), suggesting that the expression of these two miRNA might be co-regulated. These two miRNA were homologous to tca-miR-34-5p and tca-miR-277-3p. Overall, the small RNA sequencing results suggest that a subset of miRNA (~25% of all miRNA identified in adults) is differentially abundant depending on the life stage, including the aestivation.

### Inhibiting miRNA pathway changes protein levels in aestivation

**Fig. 2:**
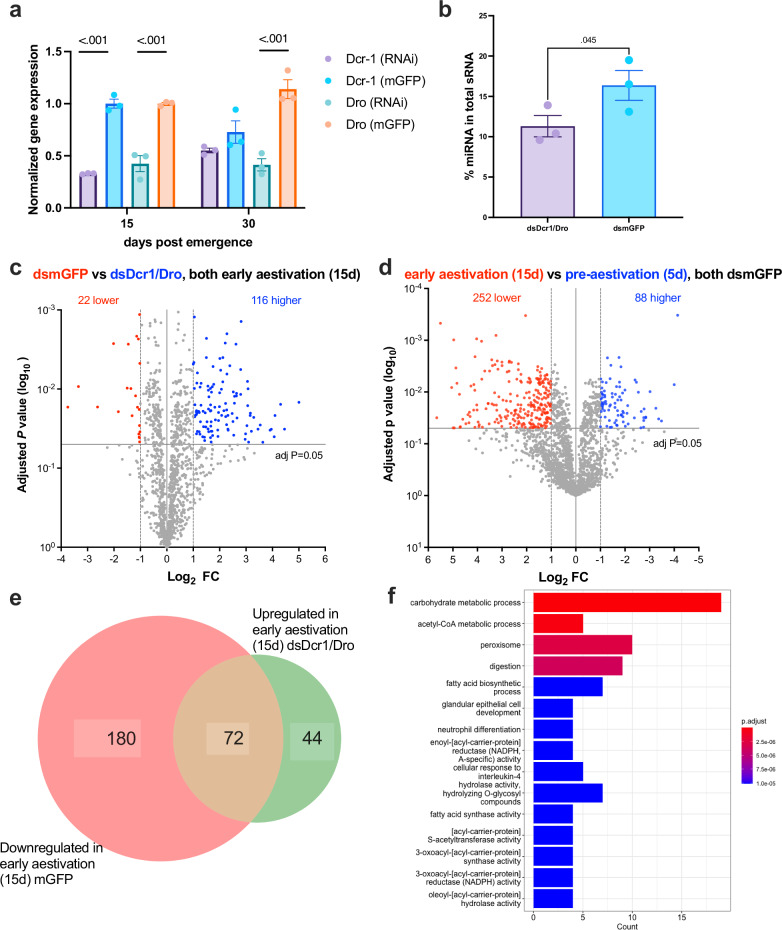
Changes at protein levels following inhibition of miRNA pathway in aestivation. **a** Normalised gene expression of *Pc-dicer-1* and *Pc-drosha* upon the feeding of chimeric dsDcr1/Dro, which simultaneously targeted both genes or control dsRNA, dsmGFP (*n* = 3, mean ± SEM, normalised to the respective gene expression in the dsmGFP group, comparison by Šídák’s test). **b** Percent miRNA in total small RNA measured through fragment analysis (*n* = 3, mean ± SEM, comparison by one-sided *t*-test). **c** Differentially abundant proteins in 15d-old (aestivating) dsDcr1/Dro-fed beetles in comparison to 15d-old dsmGFP fed beetles (*n* = 5). Proteins with log_2_ fold change (FC) higher than 1 or lower than -1 in addition to an adjusted P below 0.05 were accepted as significantly increased or decreased in abundance. The differential abundance analysis on proteins quantified through LC/MS was conducted using Perseus (v1.4). **d** Differentially abundant proteins in 15d-old dsmGFP fed beetles in comparison to 5d-old (pre-aestivating) dsmGFP fed beetles (*n* = 5). **e** Venn diagram showing the overlap between proteins with increased abundance in 15d-old dsDcr1/Dro-fed beetles (**c**) and proteins with decreased abundance in 15d-old dsmGFP fed beetles (**d**) **f** The proteins significantly increased in abundance in dsDcr1/Dro-fed beetles were subjected to gene ontology (GO) enrichment analysis using clusterProfiler (v4.1). GO terms with *P* < 0.05 were accepted as significantly enriched.

The differential abundance of miRNA during aestivation prompted us to investigate the regulatory role of the miRNA pathway during aestivation. To that end, we inhibited the miRNA pathway using a chimeric dsRNA that targeted both *dicer-1* and *drosha* (dsDcr1/Dro), which are two key nucleases involved in miRNA biogenesis. This approach was used to ensure the simultaneous inhibition of both important steps in miRNA biogenesis. We fed CSFB adults with dsDcr1/Dro or dsmGFP (control) upon adult emergence throughout the pre-aestivation period. We confirmed through RT-qPCR that both target genes were significantly knocked down on 15 days post-emergence (i.e. early aestivation) in the dsDcr1/Dro-fed CSFB compared to the control CSFB (*Pc-dicer-1:* *P* < 0.001, *t* = 7.9 and *Pc-drosha*: *P* < 0.001, *t* = 6.1 by Šídák’s test, df = 8, Fig. 2a). Also, the expression of *Pc-drosha*, but not *Pc-dicer-1*, was significantly knocked down on 30 days post-emergence (*Pc-dicer-1:* *P* = 0.133, *t* = 2.1 and *Pc-drosha*: *P* < 0.001, *t* = 7.75 by Šídák’s test, df = 8, Fig. 2a). Furthermore, the proportion of miRNA within total small RNAs was significantly reduced by 31.9% at 15 days post-emergence in dsDcr1/Dro-fed CSFB compared to the control CSFB (*P* = 0.04, *t* = 2.2, df = 4 by *t*-test, Fig. 2b).

Next, we assessed the protein levels in aestivating CSFB. In total, our proteomics approach was able to capture 2216 proteins with sufficient abundance for quantification in CSFB adults. We identified 116 proteins with significantly higher abundance in dsDcr1/Dro-fed aestivating CSFB compared to control aestivating CSFB (adjusted *P* < 0.05 by Welch’s test, Fig. 2c, Supplementary Data 2). These 116 proteins likely contained both proteins directly regulated by miRNA and proteins regulated by miRNA in an indirect manner, including through miRNA-mediated regulation of other proteins within the same gene-regulatory network. In contrast, 22 proteins had lower abundance in dsDcr1/Dro-fed aestivating CSFB (adjusted *P* < 0.05 by Welch’s test). Such a ratio—five times more proteins with higher abundance in dsDcr1/Dro-fed aestivating CSFB compared to control aestivating CSFB—was expected because reduction in miRNA-mediated repression should increase levels of proteins normally repressed by miRNA. In contrast, the proteins that decrease in abundance either reflect rare cases where miRNA positively regulates a protein or represent an indirect effect, such as compensatory regulation due to the inhibition of the miRNA pathway. Among the 116 proteins showing higher abundance in dsDcr1/Dro-fed CSFB, more than half (72 proteins, 62%) also showed a significant decrease in abundance in aestivation compared to the pre-aestivation in the control CSFB (Fig. 2d, e). This overlap shows that our RNAi approach mostly inhibited the suppression (i.e. increased the abundance) of proteins that are suppressed for aestivation in intact CSFB. In contrast, we observed no overlap between proteins with lower abundance in control aestivating CSFB compared to pre-aestivation control CSFB and those with lower abundance in dsDcr1/Dro-fed aestivating CSFB compared to control aestivating CSFB. In other words, none of the proteins downregulated during aestivation in intact beetles showed reduced levels upon the inhibition of the miRNA pathway. This finding highlights the specificity of RNAi-mediated suppression of the miRNA pathway, combined with proteomics, in identifying miRNA-regulated proteins. Overall, these results suggest that the inhibition of the miRNA pathway mostly deregulated proteins that are suppressed during aestivation, which enabled us to further investigate the role of miRNA in aestivation.

The GO enrichment analysis on proteins with higher abundance in dsDcr1/Dro-fed aestivating CSFB compared to the control aestivating CSFB showed that the deregulated proteins were mainly related to metabolic pathways, including carbohydrate and lipid metabolism and peroxisome activity (Fig. 2f, see Supplementary Data 2 for details). Furthermore, a catabolism-related term, namely digestion, was also among the most enriched pathways in the proteins increasing in abundance in dsDcr1/Dro-fed aestivating CSFB. These terms suggest that miRNA may have an impact on various aspects of the aestivation phenotype, including energy storage (e.g. lipid reserves), metabolic rate and feeding.

### Characterising the mode of regulation by miRNA in aestivation

**Fig. 3:**
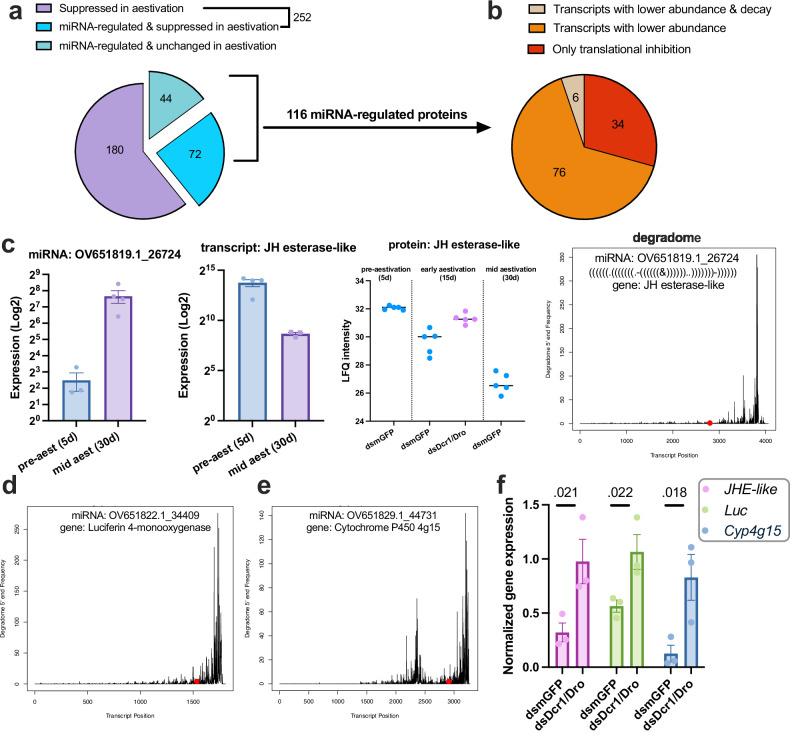
Characterising the mode of regulation by miRNA pathway in aestivation. **a** Proteomics analysis comparing aestivation and pre-aestivation in control CSFB adults identified 252 proteins with lower abundance in aestivation. Out of these 252 proteins, 72 proteins were found to be deregulated upon the RNAi of miRNA pathway in aestivation (Fig. 2), while 44 proteins were also deregulated upon the inhibition of miRNA pathway in aestivation, but they did not have lower abundance in aestivation in comparison to pre-aestivation in control CSFB. **b** Among the 116 proteins that were deregulated upon the inhibition of miRNA pathway, 82 (72 + 6) of them were found to have a lower abundance in aestivation compared to the pre-aestivation in our previous RNA-seq data^6^. We only found evidence for transcript decay for 6 out of 82 of these genes. The remaining 34 proteins only had evidence for being deregulated upon the inhibition of miRNA pathway. **c** Example of a miRNA regulated gene annotated as *juvenile hormone (JH) esterase-like*. miRNA OV651819.1_26724 is overexpressed during mid-aestivation (30d-old) in intact beetles (adjusted *P* < 0.05, *n* = 3, mean ± SEM, see Fig. 1a). The transcript level of *JH esterase-like* is downregulated in mid-aestivation in intact beetles (adjusted *P* < 0.05, *n* = 3, mean ± SEM, data from ref. ^6^). The protein level of JH esterase-like is downregulated in early (15d-old) and mid-aestivation in control beetles, while it is upregulated upon the RNAi of *dcr-1* and *dro* (dsDcr1/Dro) in early aestivation (adjusted *P* < 0.05, *n* = 5, mean ± SEM, see Fig. 2c). RNA degradome suggests the exonucleolytic decay of the *JH esterase-like* transcript (20 late pre-aestivation beetles were pooled into *n* = 1). The binding site of miRNA OV651819.1_26724 is indicated with a red dot and the complementarity between miRNA and target site is shown with dot-bracket notation. The plot was generated using CleaveLand4 and shows the degradome 5′ end frequency identified on the mRNA sequence. **d**, **e** Two other examples of transcripts decreasing abundance due to transcript decay mediated by miRNA increasing in abundance in mid-aestivation compared to pre-aestivation. **f** RT-qPCR was performed to assess the effect of dsDcr1/Dro in 15d-old beetles on the expressions of three transcripts found to be degraded by miRNA (mean ± SEM, *n* = 3, comparison by one-sided *t*-test).

Following the identification of miRNA-suppressed proteins in aestivation, we sought to characterise the mode of regulation by miRNA. Transcripts encoding 82 (76 + 6 transcripts shown in Fig. 3a, b) out of the 116 proteins showing higher abundance in dsDcr1/Dro-fed aestivating CSFB also had lower abundance in aestivation compared to the pre-aestivation (Fig. 3a, b, RNA-seq data from ref. ^6^). This suggests that most (71%) of the proteins suppressed through miRNA in aestivation were regulated at the mRNA level. Nonetheless, transcripts encoding 34 proteins with higher abundance in dsDcr1/Dro-fed aestivating CSFB did not change in abundance in aestivation, suggesting these proteins are regulated via translational inhibition (Fig. 3b). Alternatively, these 34 proteins might be regulated at the protein level such as decrease in turn-over rate.

miRNA target prediction using miRanda and 3′ UTR regions of transcripts suggested that 693 out of 3223 transcripts with lower abundance during aestivation were targeted by at least one miRNA with higher abundance in aestivation (Supplementary Fig. 1a, Supplementary Data 3). On average, each miRNA targeted 92.93 transcripts, while each transcript was targeted by an average of 2.01 miRNA among these predicted miRNA-transcript interactions (Supplementary Fig. 1b). Focusing on transcripts encoding proteins with higher abundance upon miRNA inhibition, we found that 49 transcripts (42% of the 116 proteins) could be linked to at least one miRNA with higher abundance in aestivation through target prediction (Supplementary Fig. 1c). In these cases, the average number of target transcripts per miRNA was 7.9, and each transcript was targeted by an average of 2.43 miRNA (Supplementary Fig. 1d). A similar analysis was conducted for the opposite relationship—miRNA with lower abundance in aestivation and their predicted target transcripts with higher abundance in aestivation. This analysis identified 1171 out of 3013 transcripts with higher abundance during aestivation as potential miRNA targets (Supplementary Fig. 1a). On average, each miRNA targeted 176.8 transcripts, and each transcript was targeted by an average of 3.3 miRNA within these interactions (Supplementary Fig. 1b).

Next, we performed transcriptome-wide RNA degradome sequencing in 10- and 15-day-old CSFB, representing late pre-aestivation and early aestivation stages, to investigate whether miRNA with higher abundance in aestivation (Fig. 1b, c, see Supplementary Data 1) contribute to the degradation of transcripts encoding miRNA-suppressed proteins (Fig. 2c, see Supplementary Data 2). Analysis of degradome plots provided evidence for miRNA-mediated decay of only six transcripts (Fig. 3c, d and Supplementary Figs. 2 and 3, see Supplementary Data 4 for details). In these transcripts, we observed a high frequency of degradation at the 3′ ends, suggesting a regulatory mechanism involving deadenylation followed by exonucleolytic decay, commonly observed in animals, rather than site-specific cleavage, which is typical in plants^35^ (Fig. 3c, d). Furthermore, most evidence of transcript decay was observed in the late pre-aestivation stage (10-day-old CSFB; six transcripts), compared to early aestivation (15-day-old adults; two transcripts), suggesting that transcript degradation predominantly occurs before aestivation. Notably, when analysing the entire RNA degradome dataset—beyond the scope of aestivation-related genes and miRNA—we identified four potential instances of transcript target site cleavages mediated by complementary miRNA (categorised as ‘0’ by CleaveLand4, Supplementary Fig. 4). This finding suggests that miRNA-mediated target site cleavage may still occur in CSFB, although no such evidence was found when focusing specifically on aestivation-related regulation.

The transcripts with evidence for miRNA-mediated decay included genes that are likely important for aestivation, such as *juvenile hormone (JH) esterase-like* and *cytochrome P450 (4g15)*—both of which have been previously implicated in aestivation (Fig. 3c, d)^6^. Together with our degradome sequencing data, we provide comprehensive evidence supporting the miRNA-mediated regulation of aestivation-related genes. For example, the miRNA OV651819.1_26724 is significantly overexpressed during mid-aestivation (30-day-old) in intact CSFB (Fig. 3c), while the complementary *JH esterase-like* transcript is downregulated during mid-aestivation in intact CSFB (adjusted *P* < 0.05, *n* = 3; data from^6^). Furthermore, at the protein level, JH esterase-like is downregulated in dsmGFP-fed control CSFB during early (15-day-old) and mid (30-day-old) aestivation compared to pre-aestivation (5-day-old), whereas it is upregulated in dsDcr1/Dro-fed CSFB relative to dsmGFP-fed CSFB during early aestivation (adjusted *P* < 0.05, *n* = 5; Fig. 3c). Lastly, RNA degradome analysis indicates decay of the *JH esterase-like* transcript (Fig. 3c). Gene expression measurements confirmed that the inhibition of the miRNA pathway results in the upregulation, i.e. derepression, of 3 transcripts (Fig. 3c–e) revealed by the degradome analysis in 15-day-old CSFB (*P* < 0.05, *t* = 2.94–3.13 by *t*-test, df = 4, Fig. 3f). These findings collectively suggest that miRNA play a crucial role in regulating a subset of genes involved in aestivation via post-transcriptional regulation.

### Regulation by miRNA pathway is important for the aestivation phenotype

**Fig. 4:**
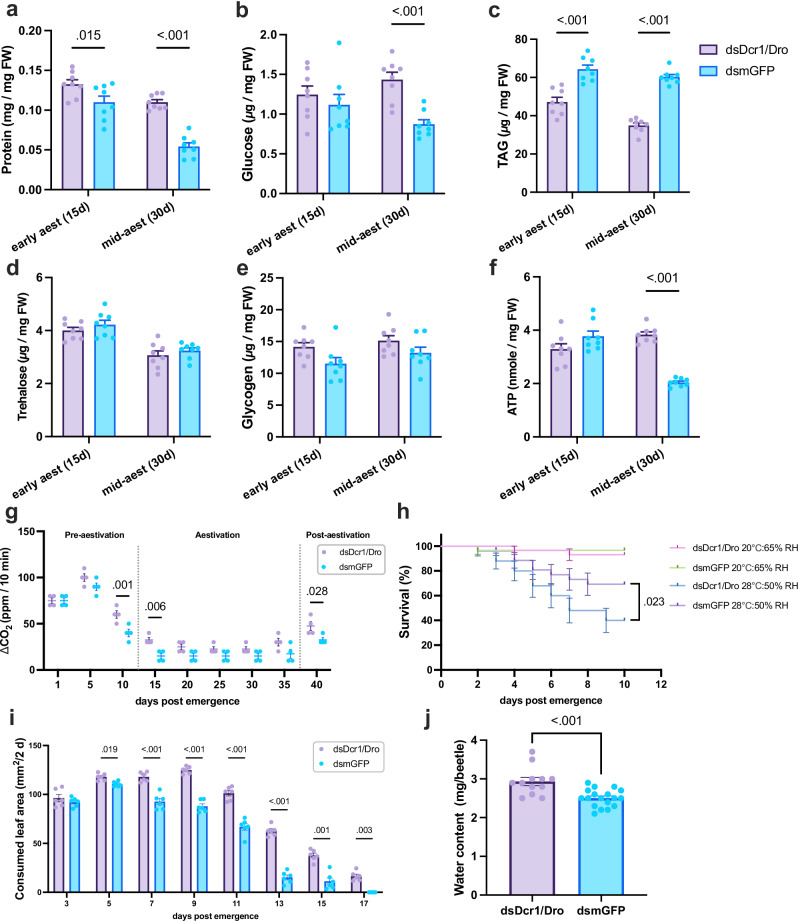
Inhibition of miRNA pathway weakens the aestivation phenotype. The miRNA pathway of CSFB adults was inhibited through feeding of chimeric dsDcr1/Dro and compared with the dsmGFP-fed control CSFB. Protein (**a**), glucose (**b**), TAG (**c**), trehalose (**d**), glycogen (**e**) and ATP (**f**) levels in whole bodies of 15 (early aestivation) and 30 (mid-aestivation) days old adults were measured. **g** The CO_2_ production was measured in CSFB post adult emergence with 5 day intervals (*n* = 4). **h** Survival analysis with aestivating CSFB (*n* = 30 mixed-sex adults, plots are Kaplan-Meier survival curves) under regular rearing conditions (20 °C and 65% relative humidity) or summer conditions (28 °C and 50% relative humidity). The survival of two dsRNA treatment groups receiving the same rearing condition treatment was compared using one-sided log-rank test. **i** Consumed leaf area was measured at 2-day intervals in two dsRNA treatment groups. **j** Water content measured in mid-aestivation (30 days) CSFB whole bodies, and a unpaired *t*-test was conducted. Two-way ANOVA (factors: day and RNAi treatment) followed by Šídák’s multiple comparisons test was performed for (**a**–**g**) and (**i**). Mean ± SEM values are provided in each panel and *P* < 0.05 are indicated. Statistical tests are two-sided unless stated otherwise.

The finding that the inhibition of the miRNA pathway disrupts the regulation of proteins in aestivation prompted us to investigate whether the aestivation is weakened at the phenotypic level upon the inhibition of the miRNA pathway. First, we performed body composition analysis on dsDcr1/Dro-fed and control CSFB at 15 days post-emergence and 30 days post-emergence, representing early aestivation and mid-aestivation, respectively. We found that overall protein content was higher in dsDcr1/Dro-fed CSFB compared to the control group on both 15- and 30-days post-emergence (*P* = 0.15 and <0.01, *t* = 2.88 and 7.07 by Šídák’s test, df = 28, Fig. 4a). Similarly, glucose content was higher in the dsDcr1/Dro-fed CSFB, albeit only on 30 days post-emergence (*P* < 0.01, *t* = 3.9, by Šídák’s test, df = 28 Fig. 4b). In contrast, TAG levels showed an opposite pattern where dsDcr1/Dro-fed CSFB had less TAG compared to the control CSFB on both 15- and 30-days post-emergence (*P* < 0.01 and <0.01, *t* = 6.4 and 9.5 by Šídák’s test, df = 28, Fig. 4c). We did not observe any significant changes in the trehalose and glycogen levels (*P* > 0.05, *t* < 2 by Šídák’s test, df = 28, Fig. 4d, e), indicating that inhibition of miRNA results in specific changes in body composition, rather than complete alteration. These results, along with the enrichment of metabolism-related proteins following miRNA pathway inhibition, suggest that miRNAs are involved in maintaining a body composition appropriate for aestivation.

It is well described that diapausing animals notably reduce their metabolic rate to maintain energy homoeostasis^36^. The ATP measurements showed that dsDcr1/Dro-fed CSFB had significantly higher ATP content than the control group on 30-days post-emergence (*P* < 0.01, *t* = 8.3 by Šídák’s test, df = 28, Fig. 4f), but not on 15-days post-emergence (*P* = 0.067, *t* = 2.2 by Šídák’s test, df = 28). In addition, we measured CO_2_ production (VCO_2_) as a proxy for the temporal changes in metabolic rate. Notably, on both 10- and 15-days post-emergence, the dsDcr1/Dro-fed CSFB had significantly higher VCO_2_ than the control CSFB (*P* < 0.01 and <0.01, *t* = 4.1 and 3.6 by Šídák’s test, df = 54, Fig. 4g). These two time points correspond to the strong decrease in the metabolism while CSFB is entering aestivation. Although both measurements supported the idea that regulation by miRNA is important for suppressing metabolism during aestivation, the effect of miRNA inhibition on ATP seems to be delayed compared to its effect on VCO_2_.

Survival experiments were conducted under two conditions, namely 28 °C temperature and 50% relative humidity (RH) and 20 °C and 65% RH (laboratory rearing conditions) and initiated with 20-day-old CSFB to investigate the previously described higher resilience of aestivating beetles to hot and dry conditions^6^. The dsDcr1/Dro-fed CSFB had the same survival rate as the control group under laboratory rearing conditions (*P* = 0.55, *χ*2 = 0.35 by logrank test, *n* = 30, Fig. 4h), meaning that inhibition of miRNA does not have a detrimental effect on the survival of CSFB adults under normal circumstances. Interestingly, however, dsDcr1/Dro-fed aestivating CSFB had significantly less survival than the control aestivating CSFB under hot and dry conditions (*P* = 0.023, *χ*2 = 4.03 by logrank test, *n* = 30, Fig. 4h). These results suggest that the regulation by the miRNA pathway is important for the resilience of aestivating CSFB to hot and dry conditions.

A defining characteristic of aestivation in CSFB, like in other insects, is the suppression of feeding activity and other activities in general. Here, we assessed whether aestivating CSFB diverged from control group in terms of feeding and activity patterns. In control CSFB, the feeding activity peaks 5 days post adult emergence and gradually declines until complete cessation of feeding activity by 15 days post-emergence (Fig. 4i). The dsDcr1/Dro-fed CSFB showed higher feeding activity compared to the control CSFB following 3-days post adult emergence until 17-days post adult emergence (*P* < 0.05, *t* > 4 by Šídák’s test, df = 5–10). Also, the movement activity measurements showed that dsDcr1/Dro-fed CSFB resumed movement activity prematurely (Supplementary Fig. 5). Specifically, the miRNA-compromised CSFB moved significantly more on days 25 and 35, representing the mid and last days of the aestivation, compared to the control CSFB (*P* = 0.016 and 0.043, *t* = 3.0 and 2.7 by Šídák’s test, df = 90, Supplementary Fig. 5). These results suggest that miRNA contributes to the activity inhibition in CSFB aestivation.

Water content spontaneously decreases in aestivation under normal rearing conditions in CSFB and some other insects, likely due to reduced water intake through feeding and increased dry body mass due to accumulated energy stores. Hence, we previously described the decrease in water content as an aestivation phenotype in CSFB^6^. Here, water content significantly increased in dsDcr1/Dro-fed CSFB during aestivation (*P* < 0.01, *t* = 3.99, by unpaired *t*-test, df = 28, Fig. 4j). Hence, the increase in water content also represents a weakened aspect of the aestivation phenotype due to the inhibition of the miRNA pathway.

Overall, the inhibition of the miRNA pathway weakened the aestivation phenotype at physiological and behavioural levels, supporting the hypothesis that miRNA functionally regulates aestivation in CSFB.

## Discussion

Insect diapause involves major changes at the gene expression level, requiring dedicated gene regulatory mechanisms^37–39^. Despite the discovery of key transcriptional factors required for diapause, such as FoxO^39,40^, a comprehensive picture of gene regulatory mechanisms involved in diapause is lacking. Here, we provide multifaceted evidence for the miRNA pathway being a key regulator of gene expression required for a successful aestivation (i.e. summer diapause) in the CSFB by combining small RNA-seq, mRNA-seq, proteomics, RNA degradome-seq and functional studies through RNAi. Key factors that enabled our approach were the robust systemic RNAi response^41^ and the obligatory nature of aestivation in CSFB^6,42^. Obligatory aestivation does not require manipulation of the rearing conditions to force insects into the aestivation programme, meaning that the molecular readouts can be readily attributable to the aestivation programme itself rather than the confounding effects of changing rearing conditions. This is important for identifying the subtle effects of miRNA on the aestivation phenotype upon the experimental inhibition of the miRNA pathway.

miRNA can regulate the abundance of a protein by causing mRNA decay and/or translational repression in animals^10,43^. Here, we found that the inhibition of the miRNA pathway mostly resulted in deregulation (increased abundance) of proteins with reduced transcript abundance in aestivation. The most parsimonious explanation is that the abundance of these proteins is regulated via mRNA decay. However, it cannot be excluded that the regulation of these proteins involved both mRNA decay and translational repression mechanisms by miRNA. Our RNA degradome analysis provided evidence for miRNA-associated decay of six transcripts, which was a very small proportion of all the miRNA-regulated proteins. RNA degradome analysis in animals is not as well-established as in plants^44,45^. To our knowledge, comprehensive degradome data from invertebrates are available only for *Caenorhabditis elegans*^46^, and we lack comparable data to define how mRNA decay occurs in beetles. Hence, further advances in RNA degradome sequencing, including both experimental and bioinformatic improvements, in non-model animals may enable further mechanistic insights and confirmation of more mRNA decay events. Moreover, our study did not assess the potential influence of spatiotemporal expression dynamics of miRNA^47^ on aestivation, as our approach relied on inhibiting the miRNA biogenesis pathway systemically. It is likely that tissue-specific and precisely timed expression of certain miRNA play an important role in the regulation of aestivation, because diapause itself is a dynamic stage^29,37^.

Interestingly, we identified 22 proteins that decreased in abundance upon the inhibition of the miRNA pathway. The decrease in abundance upon miRNA pathway inhibition suggest that these proteins are negatively regulated by other proteins that are suppressed by miRNA. Alternatively, these proteins could be directly positively regulated through miRNA-mediated increase in mRNA stability or enhanced translation^48,49^. Although most of these proteins positively regulated via miRNA were uncharacterised proteins, the ones annotated by our database included a histone protein, HIST1H1A, (Supplementary Data 2) whose upregulation might be important for overall gene regulation during aestivation^50^. Further studies are required to elucidate the mechanism behind this miRNA-mediated positive protein regulation and the functions of these proteins in aestivation.

In addition to demonstrating that numerous proteins are suppressed via the miRNA pathway, our RNAi experiments confirmed that miRNA-mediated regulation is essential for the majority of aestivation-associated phenotypes in CSFB. For instance, metabolism was identified as a main target of miRNA in this study, and the suppression of metabolism was compromised upon the RNAi of the miRNA pathway. Lower metabolic rates, in turn, are known to increase resilience to desiccation by allowing a reduction in spiracle activity^51,52^, explaining why miRNA-inhibited CSFB was less resilient to hot and dry rearing conditions. Likewise, another target of miRNA was digestion and the silencing of the miRNA pathway compromised the CSFB’s ability to cease feeding activity, a major feature of aestivation and diapause in general^53–55^. The observed increase in feeding and movement activity may be linked to a protein belonging to TRAFAC class myosin-kinesin ATPase superfamily that showed the highest increase in abundance upon the inhibition of the miRNA pathway (Supplementary Data 2). Despite many impacts of miRNA pathway inhibition on the aestivation, we did not observe any shift in the timing of aestivation initiation or termination based on the metabolic rate curves (Fig. 4g), suggesting miRNA act only to modulate the aestivation in a downstream manner, rather than determine its timing. A limitation of the RNAi approach used in this study is that only a single dsRNA construct targeting the miRNA pathway was employed. Although dsRIP web platform^56^ was used to minimise bioinformatically predicted off-target effects, the possibility of unintended off-target influences on the observed phenotypes cannot be entirely excluded. Future studies using multiple, non-overlapping dsRNA constructs targeting different components of the miRNA pathway will be essential to validate the specificity of the observed phenotypes and to rule out potential false positives.

Moreover, although the affected biological pathways in proteomics and results of the functional analyses upon miRNA inhibition were aligned, it remained mostly unclear the function of individual genes that were suppressed by miRNA. For instance, a gene annotated as *JH esterase-like* was a clear target of the miRNA during aestivation confirmed by proteomics, RNA-seq, degradome and RT-qPCR approaches (Fig. 3). As JH levels are typically reduced during diapause^50^, it is likely that the identified miRNA target *JH esterase-like* does not perform the canonical JH degrading function of JH esterases during aestivation. Further studies are required to assess the function of such genes regulated by miRNA during aestivation.

Our study raises an interesting evolutionary question: Why has the miRNA gained this important function in the regulation of diapause? We argue that miRNA is an excellent fit for mediating a significant portion of the regulation required during diapause. First, usually, more genes have to be repressed rather than upregulated during diapause^6,38,57,58^, and repression is the dominant mode of regulation mediated by miRNA^59,60^. Second, diapausing insects often need to temporally fine-tune the expression of target genes^38,61^, which is a feature of miRNA-mediated regulation, rather than completely inhibiting their expression, which is often required for cellular differentiation and involves more robust epigenetic changes such as histone modifications^62^. It should be noted that miRNA are not the only important regulators of aestivation, as only 29% (72 out of 252, Fig. 2e) of aestivation-regulated proteins were deregulated upon the inhibition of the miRNA pathway. Rather, miRNA likely work in tandem with diapause-related transcription factors such as FoxO^63^, which increase the transcription rate of genes involved in fat hypertrophy and survival. Recent studies in other insects also suggested that miRNA could be important for diapause regulation by showing differential expression of miRNA during diapause^28,29^. Interestingly, one of the miRNA found to be specifically upregulated during aestivation in this study and homologous to tca-miR-277, was also reported to be overexpressed during diapause compared to diapause termination in the seven-spot ladybird beetle, *Coccinella septempunctata*^30^. This suggests that certain miRNA may play an evolutionarily conserved role in regulating insect diapause. However, more comprehensive studies are required in other insect species to gain further evolutionary insights into the regulation of diapause through miRNA.

Climate models predict an ever-warming globe^64^, and consequently increases in pest pressure on crops due to shortened generation time have already been reported^65–67^. It is reasonable to argue that insect pests with pre-adaptations such as the aestivation observed in the CSFB might thrive under rising temperatures due to the existence of a genetic tool kit for becoming resilient to high temperatures^68,69^. This study showed that miRNA is a key pathway involved in regulating aestivation. Hence, studying potential changes in the miRNA pathway as temperatures rise may provide insights into whether and how these species adapt to high temperatures. This, in turn, could improve the accuracy of predicting which pest species will be more important in the future climate.

The ban on neonicotinoids and the emergence of pyrethroid-resistant populations have made the management of CSFB highly challenging, which necessitates the development of next-generation pest management strategies, such as RNAi^2,41,70^. The development of such strategies, in turn, requires a deep understanding of the target pest biology at the molecular level. Insights into the miRNA-mediated regulation in CSFB may support the development of innovative pest control strategies, such as those based on miRNA antagonists, to disrupt the aestivation stage of CSFB and break the pest cycle.

In summary, our study demonstrates that the microRNA pathway is a central regulatory mechanism underlying obligatory aestivation in CSFB. By integrating small RNA-seq, mRNA-seq, degradome-seq, quantitative proteomics and functional RNAi experiments, we provide robust evidence that miRNA suppress a specific subset of transcripts and proteins essential for establishing key aestivation traits. Notably, we show that the miRNA pathway plays a critical role in metabolic suppression and in conferring resilience to heat stress. Furthermore, the methodological approaches established in this study, including degradome sequencing and proteomics following miRNA pathway inhibition in a non-model insect, can serve as a valuable framework for uncovering and validating miRNA functions in the life cycles of other non-model insect species.

## Materials and methods

### Insects

The cabbage stem flea beetles (CSFB, *Psylliodes chrysocephala*) from our laboratory colony were maintained at 20 °C and 65% relative humidity under a 16:8 light/dark regime on fresh oilseed rape plants. Newly emerged adults were collected daily from pupation boxes.

### Small RNA sequencing

Total RNA was isolated from pre-aestivation (5-day old), aestivation (30-day old) and post-aestivation (55-day old) females using the Quick-RNA Tissue Kit (Zymo Research), and stored at –80 °C. The same RNA samples were previously used in our mRNA-seq study^6^ to ensure concordant data. Thawed RNA samples were analysed through fragment analysis using Agilent 2100 Bioanalyzer (Agilent Technologies) to confirm integrity before library preparation. Libraries were prepared from 6 µL input RNA using the NEBNext® Multiplex Small RNA Library Prep Kit for Illumina® (NEB, catalogue no: E7560S). Libraries were indexed through 13 PCR cycles, pooled by concentration and size-selected (135–150 bp) on a 6% polyacrylamide gel. The extracted libraries were re-evaluated through fragment analysis and sequenced by BGI Tech Solutions Co. Ltd (Hong Kong) on a DNBSEQ-G400 platform (PE50 + 5 + 10 mode). Small RNA sequencing experiments were repeated four times.

### De novo miRNA prediction

The raw sRNA-seq data underwent quality analysis using FastQC (https://www.bioinformatics.babraham.ac.uk/projects/fastqc), and low-quality (*q* < 30) reads and adaptor sequences were removed using TrimGalore! (v0.6, https://github.com/FelixKrueger/TrimGalore). All cleaned reads were combined for de novo miRNA prediction using miRDeep2 in default mode^71^. The CSFB reference genome (GenBank: GCA_927349885.1) was retrieved from NCBI for the mapper function, and mature miRNA sequences from Tribolium castaneum obtained from miRbase^72^ acted as homologous miRNA.

### RNA interference

The chimeric dsDcr1/Dro was designed to target both *Pc-dicer-1* and *Pc-drosha* using https://dsrip.uni-goettingen.de^56^. The first 210 bp was complementary to *Pc-*dicer-1, while the subsequent 208 bp was complementary to *Pc-drosha* (Supplementary Note 1). The dsDNA templates for the in vitro transcription of dsDcr1/Dro and dsmGFP (control treatment^70^) were ordered as gBlocks (IDT, Germany). We used two pairs of primers, each adding T7 promoter to either end of the dsDNA template through two separate PCR reactions using Q5 high fidelity polymerase (Supplementary Table 1). The two strands of the dsRNA were synthesised separately using MEGAscript™ T7 Transcription Kit (Thermo Fisher Scientific, Germany) according to the manufacturer’s instructions and combined in equimolar concentration. The RNA strands were first denatured at 94 °C for 5 min and subsequently annealed at room temperature for 40 min. The length of dsRNA was confirmed by 1% agarose gel electrophoresis. dsRNA was diluted to 1 µg/µL in nuclease-free water and combined with Triton-X (200 ppm final concentration, Sigma-Aldrich). 1 µL of dsRNA solution was homogenously spread on 100 mm^2^ disks punched out from young oilseed rape leaves. Freshly treated leaf disks were provided to newly emerged CSFB adults every 2 days until 17 days post-adult emergence.

### Proteomics

Whole bodies of four female CSFB treated with either dsDcr1/Dro or dsmGFP were sampled at 5 and 15 days post-adult emergence, flash-frozen in liquid nitrogen and homogenised by grinding. Additionally, four dsmGFP-treated females were sampled at 30 days post-adult emergence. Proteins from the samples were extracted using urea denaturation buffer, which contained 6 M urea, 2 M thiourea and 10 mM HEPES (pH 8.0) at a proportion of 1 ml per 100 mg homogenised sample. The supernatant containing protein was recovered through centrifugation at 12,000 × *g* for 5 min. Bicinchoninic acid assay was used to determine protein concentration. Next, proteins were precipitated using chloroform/methanol extraction^73^. The protein pellet was suspended in Rapigest SF solution (0.1%, Waters) and digested using trypsin (1:20, Serva, Germany). The digested protein was prepared for LC/MS analysis by adding TFA at a 1:10 ratio, incubating the mixture at 37 °C for 45 min and subsequently drying it using a SpeedVac. A standard C18 stage tipping protocol was performed to purify the peptides^74,75^. The proteomics experiments were repeated for a total of 5 biological replicates.

LC/MS analysis of the peptide samples was performed with an Ultimate 3000 system (Thermo Fisher Scientific) coupled to a Q Exactive HF mass spectrometer (Thermo Fisher Scientific). Peptides were separated by reversed phase liquid chromatography and on-line ionised by nano-electrospray (nESI) using the Nanospray Flex Ion Source (Thermo Fisher Scientific). Full scans were recorded in a mass range of 300–1650 m/z at a resolution of 30,000. Data-dependent top 10 HCD fragmentation was performed at a resolution of 15,000 with dynamic exclusion enabled. The XCalibur 4.0 software (Thermo Fisher Scientific) was used for LC-MS method programming and data acquisition. MaxQuant (v2.6) and Perseus (v1.6) proteomics software packages were used for data analysis. Calculation of label-free quantification values (LFQ) was performed, and a CSFB-adult-specific protein database was used that was generated from our transcriptome data^6^. Proteins with fewer than three peptide counts were discarded. Proteins with an adjusted *P* < 0.05 and either log_2_ fold > 1 or log_2_ fold < −1 were considered significantly increased or decreased in abundance, respectively.

### miRNA target prediction

The target transcripts of miRNA showing differential abundance in aestivation were predicted using miRanda using the ‘strict’ mode and 140 as miRanda score threshold. As the target input, the 3′ UTR regions from the direction corrected CSFB transcriptome was extracted using TransDecoder (https://github.com/TransDecoder/TransDecoder/wiki). Next, the transcripts showing differential abundance in aestivation^6^ were identified in the miRanda output.

### RNA degradome sequencing

We isolated total RNA from 20 intact 10- or 15-day-old CSFB female whole bodies, using the Quick-RNA Tissue kit (Zymo Research). RNA quality was assessed through fragment analysis using Agilent 2100 Bioanalyzer (Agilent Technologies), and 100 µg of RNA was used as input for degradome library preparation based on a published method^76^. Polyadenylated RNA was purified using Dynabeads mRNA Purification kit (ThermoFisher) and Oligo d(T) 25 Magnetic Beads (NEB). The 5′ RNA adaptor (100 µM, IDT, Germany, Supplementary Table 2) was ligated to RNA using T4 RNA ligase (NEB) and 5′ RNA adaptor ligated polyadenylated RNA was purified using Dynabeads mRNA purification kit (ThermoFisher). First, the cDNA strand was synthesised using ProtoScript TM II reverse transcriptase (NEB). The reaction for the second strand cDNA synthesis included first cDNA strand, MyFi Mix (BioCat), and 5′ and 3′ adaptor primers (Supplementary Table 2), and 15 PCR cycles were performed. The PCR product was purified using Monarch PCR & DNA cleanup kit and digested with EcoP151 (NEB). dsDNA adaptors (Supplementary Table 2) were ligated to the digested DNA using T4 DNA ligase (NEB). The dsDNA adaptor-ligated DNA (79 bp) was purified through PAGE and concentrated through ethanol precipitation. Thirteen PCR cycles were performed using MyFi Mix (BioCat) to add the SR1 indexes (Supplementary Table 2) to the libraries. The libraries were pooled and purified through PAGE. The sequencing service was provided by BGI Tech Solutions Co. Ltd (Hong Kong) on a DNBSEQ-G400 platform (PE50 + 5 + 10 mode). The degradome frequency plots were generated by mapping degradome reads to our CSFB adult transcriptome using CleaveLand4 (v4.5, https://github.com/MikeAxtell/CleaveLand4). The degradome frequency plots were manually investigated to find evidence for decay of mRNA showing complementary to miRNA with higher abundance in aestivation (*n* = 1 per age).

### RT-qPCR and small RNA composition

Four CSFB females receiving dsDcr1/Dro or dsmGFP treatment were flash-frozen in liquid nitrogen after wing removal. Total RNA from CSFB samples was extracted using the Quick-RNA Tissue & Insect MicroPrep™ Kit (Zymo Research, Germany). RT-qPCR was conducted with the Luna One-step RT-qPCR kit (New England Biolabs, Germany) on a 384-well plate, with three technical replicates and three biological replicates, using the CFX384 Touch Real-Time PCR system (Bio-Rad). Each well contained 0.8 μL of total RNA (500 ng/μL) and 0.8 μL of a 10 mM forward and reverse primer mix (IDT, Germany) to measure the expression of *Pc*-*dicer-1*, *Pc*-*drosha*, 3 miRNA-regulated genes and a reference gene, *Pc*-*rps4e*, optimised for RNAi experiments by Cedden et al.^41^ (primers and accessions were provided in Supplementary Table 3). The normalised expression values were obtained using CFX Maestro Software (Bio-Rad).

The total RNA extracted from 15-day-old CSFB adults were subjected to fragment analysis using Agilent 2100 Bioanalyzer (Agilent Technologies), and the miRNA percentage was calculated using the ProSize data analysis software. These experiments were repeated three times.

### Body composition

The fresh weights of individual CSFB were measured to normalise the following body content measurements. The whole bodies were frozen using liquid nitrogen and homogenised by grinding. Trehalose from the samples was measured using the Trehalose Assay Kit (Megazyme). Glucose was measured using the D-Glucose HK Assay kit (Megazyme) and separated homogenised samples were first incubated with 0.5 units of amyloglycosidase for 4 h at 37 °C to obtain glycogen content. Triglyceride content was obtained by calculating the difference between total glycerol and free glycerol for each sample using Triglyceride Colorimetric Assay Kit (Cayman) and Free Glycerol Reagent Kit (Sigma), respectively. The ATP content was measured using ATP Assay Kit (Sigma-Alrdrich). Water content was measured by calculating the difference between initial fresh weight and weight after drying at 60 °C for 72 h. Body composition analyses were repeated 8 times.

### Survival analysis

CSFB adults receiving dsDcr1/Dro or dsmGFP (*n* = 30 mixed-sex adults that were 20-day-old at the start of the experiment) were transferred into climate chambers set to 28 °C and 50% relative humidity or regular rearing conditions (20 °C and 65% relative humidity). The regular rearing conditions served as reference conditions because these were previously optimised for the rearing of CSFB adults^6^. The conditions of 28 °C and 50% relative humidity were selected to represent realistic heat stress, as they fall within the highest temperature (32 °C) and lowest humidity (39%) recorded in Berlin, Germany, in August 2023 (www.timeanddate.com/weather), while also remaining within the operational limits of our climate chambers. The survival of individual beetles was monitored daily using a fine brush; individuals that showed no movement upon stimulation were recorded as dead.

### Feeding and movement activity

The leaves were flattened beside a 1 cm reference line for calibration and photographed using a 25-megapixel camera. The remaining leaf area was calculated with ImageJ software (v1.8, https://imagej.nih.gov) using the ‘Analyze Particles’ function, and this area was subtracted from the initial leaf disk area (400 mm²).

Beetles were individually placed in vented Petri dishes (100 mm × 15 mm) and tracked using a Zantiks LT unit (Zantiks Ltd.) with a 4 × 5 layout under white light at room temperature. Movement was recorded in 5 s bins over 2 h. Total movement (mm) was summed per beetle, and non-zero bins were averaged to calculate speed (mm/s).

### Statistics and reproducibility

Cleaned small RNA reads were used to calculate raw count values of mature miRNA sequences per library using miRDeep2’s ‘quantifier’ function (*n* = 3–4). These raw counts served as input for differential expression analysis with DESeq2 (v1.46)^77^. Aestivation vs. pre-aestivation and aestivation vs. post-aestivation comparisons were conducted, and miRNA with *P* < 0.05 were accepted as differentially abundant.

Proteins with fewer than three peptide counts were discarded. Proteins with an adjusted *P* < 0.05 and either log_2_ fold > 1 or log_2_ fold < −1 were considered significantly increased or decreased in abundance, respectively (*n* = 5). The proteins with significantly higher abundance were subjected to GO enrichment analysis using R package ‘clusterProfiler’ (v3.17) and our previous functional annotation for CSFB adults generated by Trinotate (v4.0, https://github.com/Trinotate)^6^. The most significantly enriched 15 GO pathways were plotted.

The normalised expression data for each target gene was analysed using Two-way ANOVA (factors: day and treatment) followed by Šídák’s multiple comparisons test to compare the RNAi and control groups (*n* = 3). miRNA percentage values were analysed through unpaired one-sided *t*-test (*n* = 3).

Survival data was analysed using logrank test to compare the RNAi and control groups reared under different conditions (*n* = 30 per group). Body composition (*n* = 8), VCO_2_, (*n* = 4, paired) feeding (*n* = 6, paired) and movement (*n* = 10) data, which were assumed to be normally distributed, were analysed using two-way ANOVA (factors: day and treatment) followed by Šídák’s multiple comparisons test to compare the RNAi and control groups. Water content data was analysed using unpaired *t*-test.

### Reporting summary

Further information on research design is available in the Nature Portfolio Reporting Summary linked to this article.

## Supplementary information

Supplementary Information

Description of Additional Supplementary Files

Supplementary Data 1

Supplementary Data 2

Supplementary Data 3

Supplementary Data 4

Reporting Summary

## Data Availability

All source data are available on figshare at: 10.6084/m9.figshare.29378867^78^, NCBI Sequence Read Archive (BioProjects: PRJNA1196009 and PRJNA1196165), and ProteomeXchange Consortium via the PRIDE partner repository (dataset identifier PXD061878). Processed data files are available as Supplementary Data (Supplementary Data 1: de novo identification and differential abundance analysis of miRNA, Supplementary Data 2: differential abundance analysis on proteins, Supplementary Data 3: miRNA target prediction, Supplementary Data 4: RNA degradomics analysis).

## Code availability

Codes are available from Figshare: 10.6084/m9.figshare.29651558.v1^79^.
