# Supplementary material for: The microRNA pathway regulates obligatory aestivation in the cabbage stem flea beetle Psylliodes chrysocephala
Source: Commun Biol. 2025 Aug 27;8:1288. doi: 10.1038/s42003-025-08721-5 (PMC12381080; doi:10.1038/s42003-025-08721-5)
Supplement: Supplementary file 1 — Supplementary Information [file 42003_2025_8721_MOESM1_ESM.pdf]

Supplementary information for

The microRNA pathway regulates obligatory aestivation in the cabbage stem flea beetle *Psylliodes chrysocephala*

Gözde Güney[†,1], Kerstin Schmitt[2,3], Johan Zicola[4], Umut Toprak[5], Michael Rostás[1,*], Stefan Scholten[4,*] & Doga Cedden[†,1,6,*]

[1]Agricultural Entomology, Department of Crop Sciences, University of Göttingen, Göttingen, Germany

[2] Institute of Microbiology and Genetics, Department of Molecular Microbiology and Genetics, University of Göttingen, Göttingen, Germany

[3] Göttingen Center for Molecular Biosciences (GZMB), Service Unit LCMS Protein Analytics, University of Göttingen, Göttingen, Germany

[4] Division of Crop Plant Genetics, Department of Crop Sciences, University of Göttingen, Göttingen, Germany

[5]Ankara University, Faculty of Agriculture, Department of Plant Protection, Ankara, Turkey

[6] Department of Evolutionary Developmental Genetics, University of Göttingen, Johann-Friedrich-Blumenbach Institute, Göttingen Center for Molecular Biosciences, Göttingen, Germany

† These two authors have contributed equally to this work

*Doga Cedden, Stefan Scholten, Michael Rostás

Email: doga.cedden@biologie.uni-goettingen.de, stefan.scholten@uni-goettingen.de,michael.rostas@uni-goettingen.de

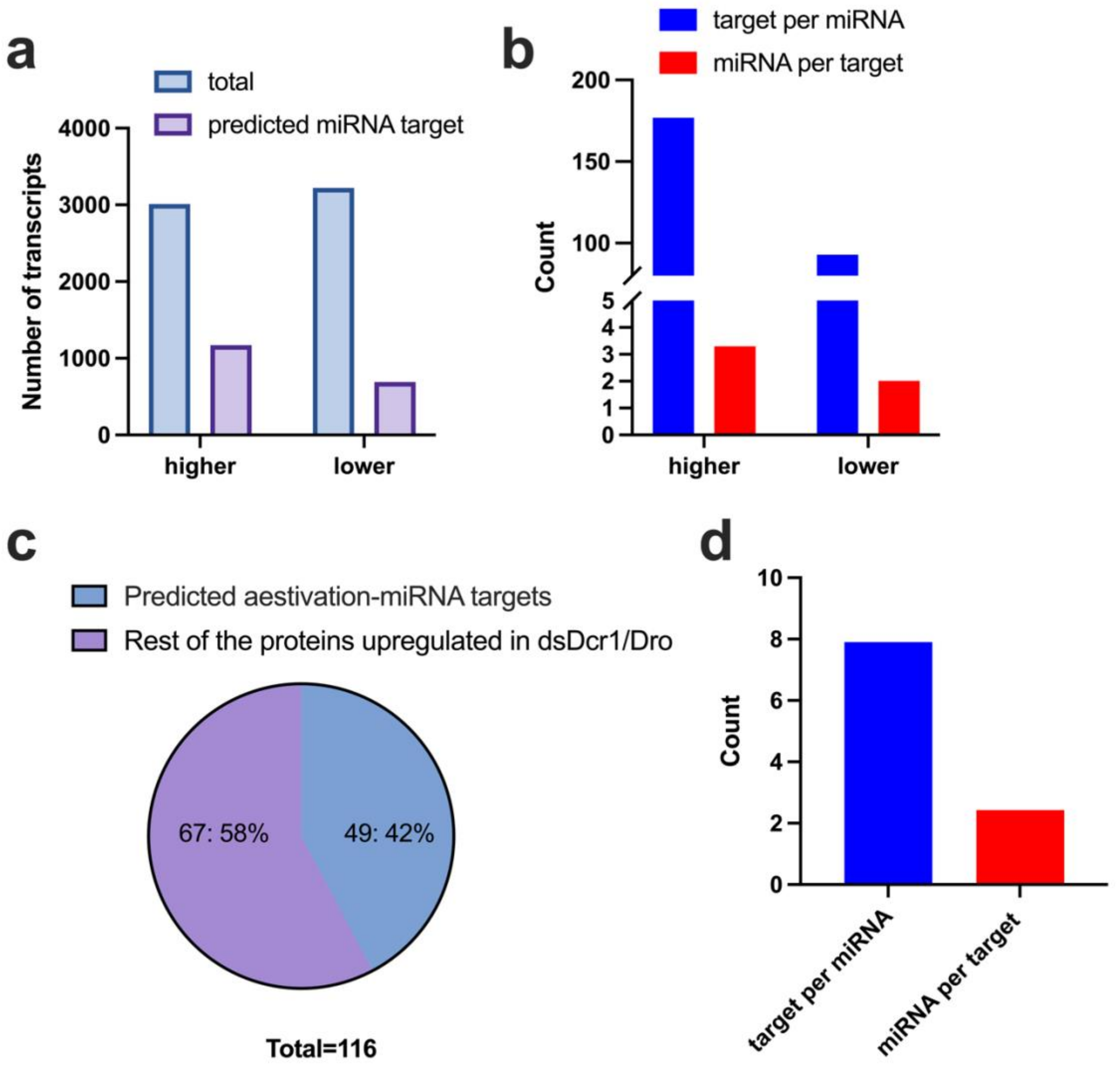

Supplementary Figure 1. Integration of miRanda target predictions with transcriptome and proteomics datasets. a) Number of total transcripts with higher or lower abundance in aestivation and the subset of these that were predicted to be targeted by at least one miRNA with reverse expression in aestivation. The target prediction was performed using miRanda and 3' UTRs of transcripts in CSFB adults. b) Counts of target transcripts per miRNA and miRNA per target transcripts among the transcripts with higher or lower abundance in aestivation. c) Pie chart indicating transcripts predicted to be targeted at least by one miRNA upregulated in aestivation and the rest of the proteins. d) Counts of target transcripts per miRNA and miRNA per target transcripts among the transcripts with lower abundance in aestivation that encode proteins determined to be negatively regulated by miRNA (see Fig. 2).

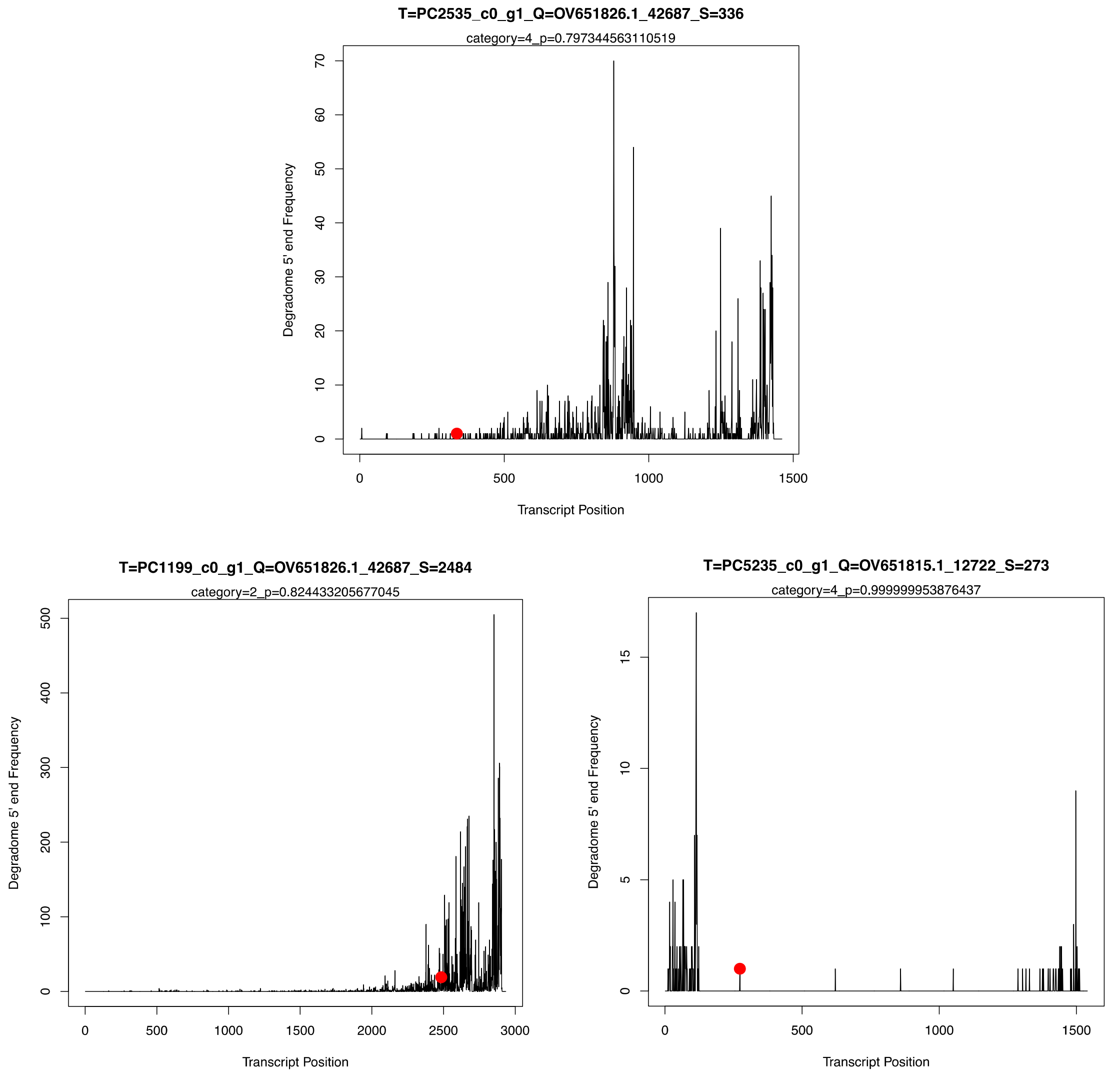

Supplementary Figure 2. Evidence for transcript decay mediated by miRNA with higher abundance in aestivation. RNA degradomics was conducted in 10-day-old cabbage stem flea beetle adults. Twenty whole bodies were pooled into one sequencing lane. Peaks indicate reads mapping to the 5' end of RNA cleavage fragments. The region complementary to the miRNA is indicated with a red dot. These data suggest the exonucleolytic decay of the transcripts.

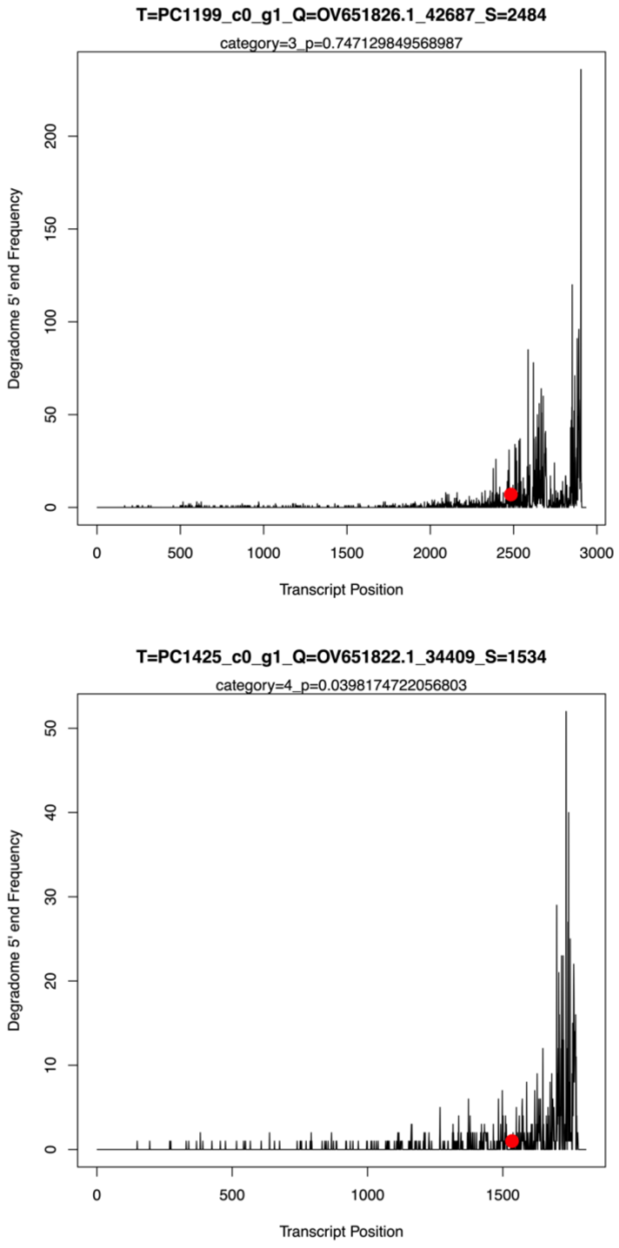

Supplementary Figure 3. Evidence for transcript decay mediated by miRNA with higher abundance in aestivation. RNA degradomics was conducted in 15-day-old cabbage stem flea beetle adults. Twenty whole bodies were pooled into one sequencing lane. Peaks indicate reads mapping to the 5' end of RNA cleavage fragments. The region complementary to the miRNA is indicated with a red dot. These data suggest the exonucleolytic decay of the transcripts.

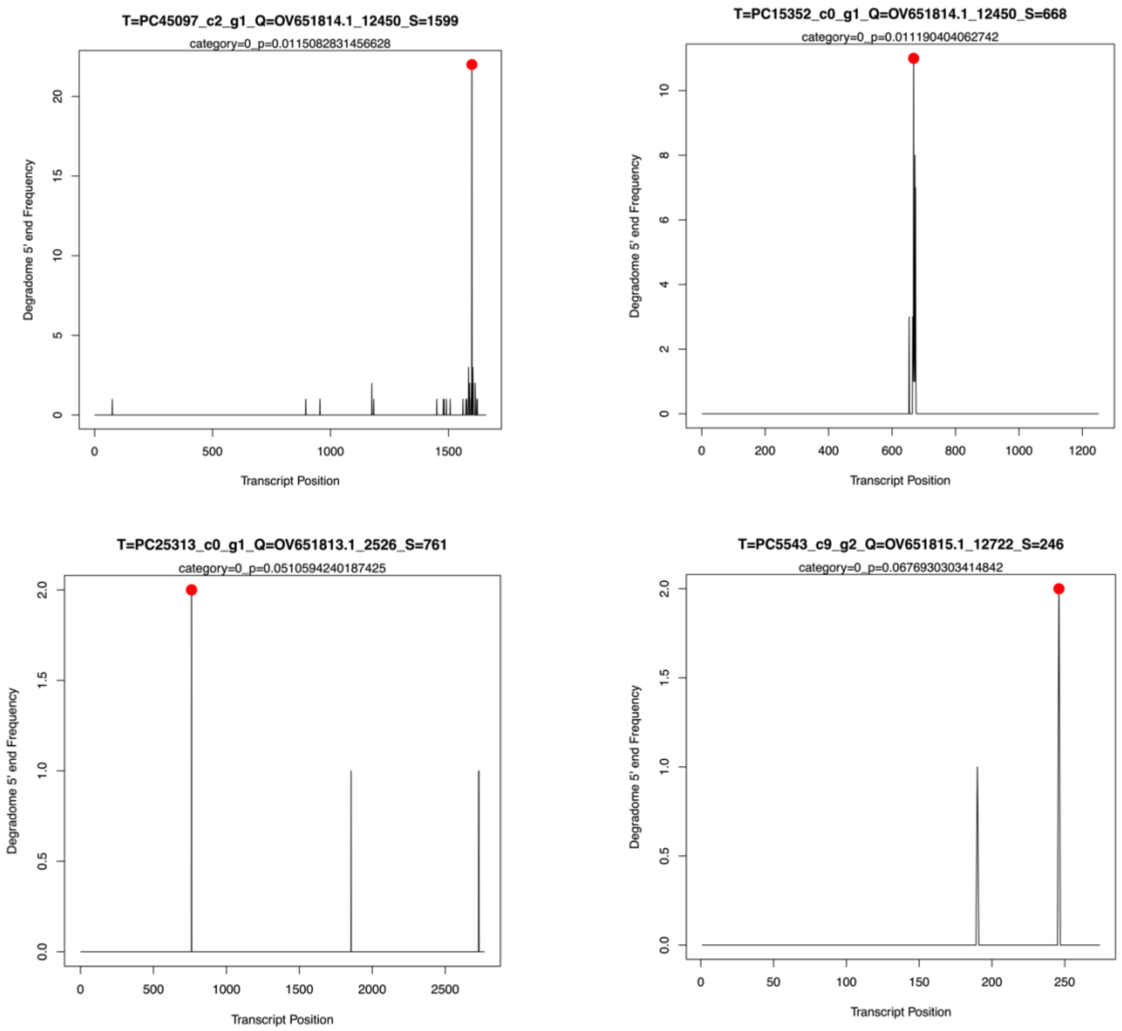

Supplementary Figure 4. Evidence for target site cleavages mediated by miRNA in the transcriptome of cabbage stem flea beetle. RNA degradomics was conducted in 10- and 15-day-old cabbage stem flea beetle (CSFB) adults. Twenty whole bodies were pooled into one sequencing lane. Plots were generated by CleaveLand4 and they were categorized as "category 0", indicating the highest level of evidence for target site cleavage. Peaks indicate reads mapping to the 5' end of RNA cleavage fragments. The region complementary to the miRNA is indicated with a red dot. However, these transcripts or miRNA were not found to be related to aestivation in our other datasets (see Fig. 1 and Fig. 2 in the main manuscript).

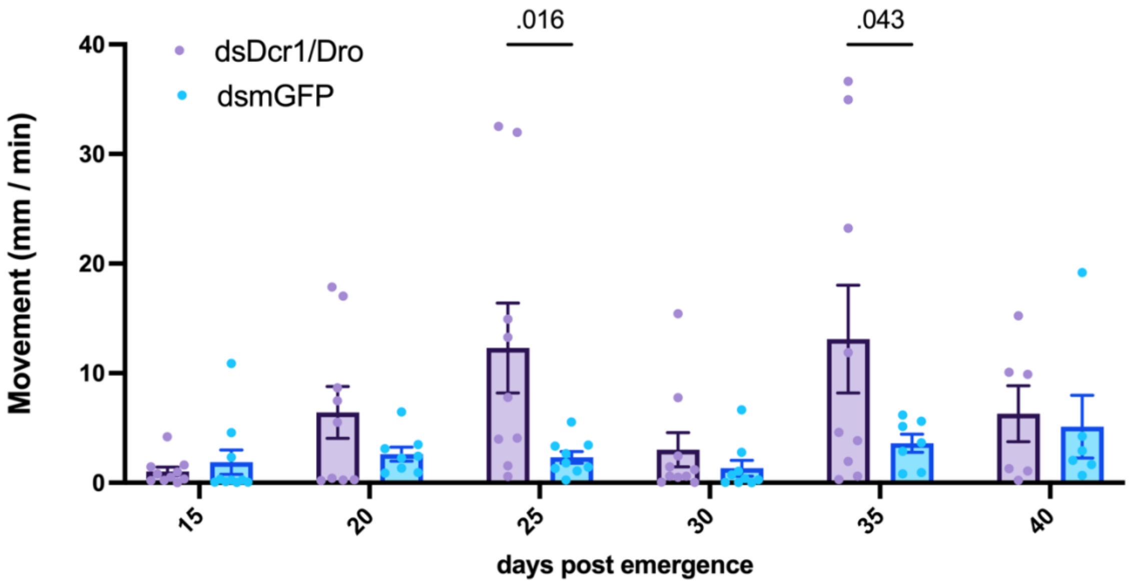

Supplementary Figure 5. Temporal movement activity measurements in CSFB adults fed with dsDcr1/Dro or dsmGFP. The movement activity was measured using Zantiks LT unit for 2 h spanning the morning activity peak and statistically analyzed using two-way ANOVA followed by Šídák's multiple comparison test (n = 6-10 per treatment, mean ± SEM). Movement is expressed as millimeters per minute (mm/min).

Supplementary Note 1. Sequence of chimeric dsDcr-1/Dro

ATCCAAGCTCATGTTCCTGGTAGCAGCCATGTCAGCGACTGACCAACAATTAGCGGGAAAT<br>TGAGCATCGATAAGAGCTTCCTCTAGTTGTTTGGGTACGTAAAAACATGGTGGTAACCAGT<br>TGTCGTGAGGATCAAATTTTGTAGCTATCATAAACTCGCCCAGATTTTTGCGCCTGCCCAAT<br>CGATATAAGTTCAAATTGCTGACTTGGCCTTTTTGGCGGCGTTCATTTCGGCTTGCTGGATA<br>CTGTGGCCCATTGCACTAGCCAGTCTCCTACCTCTAAAATACACAGCTACTGTATACACTCT<br>CGTATTAGTTGGTCCTTTACATTCAATGACCTTATAAACCGGAATATCGGGTTCGCCGCCGT<br>CCATAGTACGCAAAGTAAGGCAGCACTGCTGCAATTTCGATTTGGGAT

*The first 210 base pairs of the dsRNA target *Pc-dicer-1* (yellow) and the subsequent 208 base pairs target *Pc-drosha* (aqua). The transcriptome for CSFB adult is available on NCBI (BioProject: PRJNA930726 and TSA: GKIH00000000.1). The accessions for *Pc-dicer-1* and *Pc-drosha* are GKIH01052927.1 and GKIH01081364.1, respectively.

Supplementary Table 1. Sequences of the primers used for dsRNA synthesis through in vitro transcription

Name	Purpose	Direction	Sequence
dsmGFP	In vitro transcription	Forward	GGACCCTGACCTACGGCCTAT
dsmGFP	In vitro transcription	Reverse	GGTGCCGTCCTCGTACTAGTT
dsmGFP	In vitro transcription	Forward with T7	GAATTGTAATACGACTCACTATAGGACCCTGACCTACGGCCTAT
dsmGFP	In vitro transcription	Reverse with T7	GAATTGTAATACGACTCACTATAGGTGCCGTCCTCGTACTAGTT
dsDcr-1/Dro	In vitro transcription	Forward	ATCCAAGCTCATGTTCCTGGTAGC
dsDcr-1/Dro	In vitro transcription	Reverse	ATCCCAAATCGAAATTGCAGCAG
dsDcr-1/Dro	In vitro transcription	Forward with T7	TAATACGACTCACTATAGGATCCAAGCTCATGTTCCTGGTAGC
dsDcr-1/Dro	In vitro transcription	Reverse with T7	TAATACGACTCACTATAGGATCCCAAATCGAAATTGCAGCAG

Supplementary Table 2. Sequences of the primers and adaptors used for degradome sequencing

Name	Purpose	Direction	Sequence
5′ RNA adaptor	RNA degradome	Forward	GUUCAGAGUUCUACAGUCCGACGAUCAGCAG
RT-primer	RNA degradome	Reverse	CGAGCACAGAATTAATACGACTTTTTTTTTTTTTTTTTT
5′ adaptor	RNA degradome	Forward	GTTCAGAGTTCTACAGTCCGAC
3′ adaptor	RNA degradome	Reverse	CGAGCACAGAATTAATACGACT
dsDNA top	RNA degradome	Forward	TGGAATTCTCGGGTGCCAAGG
dsDNA bottom	RNA degradome	Reverse	CCTTGGCACCCGAGAATTCCA
Final 5′PCR primer	RNA degradome	Forward	AATGATACGGCGACCACCGAGATCTACACGTTCAGAGTTCTACAGTCCGA
Final 3′PCR primer for 10d-old CSFB	RNA degradome	Reverse with index	CAAGCAGAAGACGGCATACGAGATTTAGGCGTGACTGGAGTTCAGACGTGTGCTCTTCCGATCT
Final 3′PCR primer for 15d-old CSFB	RNA degradome	Reverse with index	CAAGCAGAAGACGGCATACGAGATGATCAGGTGACTGGAGTTCAGACGTGTGCTCTTCCGATCT

Supplementary Table 3. Sequences of the primers used for RT-qPCR

Name	Purpose	Direction	Sequence
Pc-dicer-1[1]	RT-qPCR	Forward	TCCCGATGATCAACGTAGCG
Pc-dicer-1[1]	RT-qPCR	Reverse	TATTCGGACCCAGGGAATGC
Pc-drosha[2]	RT-qPCR	Forward	ACGCAGACGAAATCAAGGGT
Pc-drosha[2]	RT-qPCR	Reverse	TGCGGTGGTCTGATTCCAAA
Pc-rps4e[3]	RT-qPCR	Forward	GGGTCGTGTTGGTACGGTAA
Pc-rps4e[3]	RT-qPCR	Reverse	AGTAGCAAACACGTGGCCAT
Pc-JHE-like[4]	RT-qPCR	Forward	CAGGTACGGCCAAGCAAGTA
Pc-JHE-like[4]	RT-qPCR	Reverse	CGTCTGGAACGTCTGGCATA
Pc-cyp4g15[5]	RT-qPCR	Forward	TCCAGCTTCGTCCTCTGTCT
Pc-cyp4g15[5]	RT-qPCR	Reverse	GCAATTACTGGCACTGGTGG
Pc-luc[6]	RT-qPCR	Forward	CCCGCAGAGCTAGAAGCAAT
Pc-luc[6]	RT-qPCR	Reverse	CTCATCAGGTGCTCCAACGA

1 Accession for Pc-dicer-1 is GKIH01052927.1 in TSA for CSFB adult: GKIH00000000.1

2 Accession for Pc-drosha is GKIH01081364.1 in TSA for CSFB adult: GKIH00000000.1

3 Accession for *Pc-rps4e* is GKIH01045819.1 in TSA for CSFB adult: GKIH00000000.1

4 Accession for *Pc-JHE-like* is GKIH01077261.1 in TSA for CSFB adult: GKIH00000000.1

5 Accession for *Pc-cyp4g15* is GKIH01067546.1 in TSA for CSFB adult: GKIH00000000.1

6 Accession for *Pc-luc* is GKIH01046683.1 in TSA for CSFB adult: GKIH00000000.1
