# Supplementary material for: The microRNA pathway regulates obligatory aestivation in the cabbage stem flea beetle Psylliodes chrysocephala
Source: Commun Biol. 2025 Aug 27;8:1288. doi: 10.1038/s42003-025-08721-5 (PMC12381080; doi:10.1038/s42003-025-08721-5)
Supplement: Supplementary file 2 — Description of Additional Supplementary Files [file 42003_2025_8721_MOESM2_ESM.pdf]

Description of Additional Supplementary Files

File name: Supplementary Data 1

Description: De novo identification and differential abundance analysis of miRNA at different life stages in the cabbage stem flea beetle

File name: Supplementary Data 2

Description: Differential abundance analysis on proteins following the inhibition of miRNA pathway

File name: Supplementary Data 3

Description: miRNA target prediction by miRanda

File name: Supplementary Data 4

Description: RNA degradomics analysis in 10- and 15-day-old cabbage stem flea beetle adults
