# Supplementary material for: The microRNA pathway regulates obligatory aestivation in the cabbage stem flea beetle Psylliodes chrysocephala
Source: Commun Biol. 2025 Aug 27;8:1288. doi: 10.1038/s42003-025-08721-5 (PMC12381080; doi:10.1038/s42003-025-08721-5)
Supplement: Supplementary file 7 — Reporting Summary [file 42003_2025_8721_MOESM7_ESM.pdf]

nature portfolio

Corresponding author(s): Doga Cedden, Stefan Scholten, Michael Rostás

Last updated by author(s): Jul 30, 2025

Reporting Summary

Nature Portfolio wishes to improve the reproducibility of the work that we publish. This form provides structure for consistency and transparency in reporting. For further information on Nature Portfolio policies, see our Editorial Policies and the Editorial Policy Checklist.

Statistics

For all statistical analyses, confirm that the following items are present in the figure legend, table legend, main text, or Methods section.

n/a	Confirmed	
☐	☒	The exact sample size (*n*) for each experimental group/condition, given as a discrete number and unit of measurement
☐	☒	A statement on whether measurements were taken from distinct samples or whether the same sample was measured repeatedly
☐	☒	The statistical test(s) used AND whether they are one- or two-sided<br>*Only common tests should be described solely by name; describe more complex techniques in the Methods section.*
☒	☐	A description of all covariates tested
☐	☒	A description of any assumptions or corrections, such as tests of normality and adjustment for multiple comparisons
☐	☒	A full description of the statistical parameters including central tendency (e.g. means) or other basic estimates (e.g. regression coefficient) AND variation (e.g. standard deviation) or associated estimates of uncertainty (e.g. confidence intervals)
☐	☒	For null hypothesis testing, the test statistic (e.g. *F*, *t*, *r*) with confidence intervals, effect sizes, degrees of freedom and *P* value noted<br>*Give P values as exact values whenever suitable.*
☒	☐	For Bayesian analysis, information on the choice of priors and Markov chain Monte Carlo settings
☒	☐	For hierarchical and complex designs, identification of the appropriate level for tests and full reporting of outcomes
☒	☐	Estimates of effect sizes (e.g. Cohen's *d*, Pearson's *r*), indicating how they were calculated

Our web collection on statistics for biologists contains articles on many of the points above.

Software and code

Policy information about availability of computer code

Data collection	XCalibur 4.0 software (Thermo Fisher Scientific)
Data analysis	TrimGalore! (v0.6), DESeq2 (v1.46), clusterProfiler" (v3.17), miRDeep2 (v0.1.3), MaxQuant (v2.6), Perseus (v1.6), CleaveLand4 (v4.5)

For manuscripts utilizing custom algorithms or software that are central to the research but not yet described in published literature, software must be made available to editors and reviewers. We strongly encourage code deposition in a community repository (e.g. GitHub). See the Nature Portfolio guidelines for submitting code & software for further information.

Data

Policy information about availability of data

All manuscripts must include a data availability statement. This statement should provide the following information, where applicable:

- Accession codes, unique identifiers, or web links for publicly available datasets
- A description of any restrictions on data availability
- For clinical datasets or third party data, please ensure that the statement adheres to our policy

All source data are available on figshare at: https://doi.org/10.6084/m9.figshare.2937886778, NCBI Sequence Read Archive (BioProjects: PRJNA1196009 and PRJNA1196165), ProteomeXchange Consortium via the PRIDE partner repository (dataset identifier PXD061878). Processed data files are available as Supplementary Data (Supplementary Data 1: de novo identification and differential abundance analysis of miRNA, Supplementary Data 2: differential abundance analysis on proteins, Supplementary Data 3: miRNA target prediction, Supplementary Data 4: RNA degradomics analysis).

Research involving human participants, their data, or biological material

Policy information about studies with human participants or human data. See also policy information about sex, gender (identity/presentation), and sexual orientation and race, ethnicity and racism.

Reporting on sex and gender	This information has not been collected.
Reporting on race, ethnicity, or other socially relevant groupings	This study did not involve human participants.
Population characteristics	This study did not involve human participants.
Recruitment	This study did not involve human participants.
Ethics oversight	This study did not involve human participants.

Note that full information on the approval of the study protocol must also be provided in the manuscript.

Field-specific reporting

Please select the one below that is the best fit for your research. If you are not sure, read the appropriate sections before making your selection.

☒ Life sciences ☐ Behavioural & social sciences ☐ Ecological, evolutionary & environmental sciences

For a reference copy of the document with all sections, see nature.com/documents/nr-reporting-summary-flat.pdf

Life sciences study design

All studies must disclose on these points even when the disclosure is negative.

Sample size	No statistical methods were used to pre-determine sample size. Omics experiments in the study incorporated commonly used sample sizes in the literature (n = 3 for RNA-seq and n = 5 for proteomics).
Data exclusions	No data were excluded.
Replication	Key experiments were replicated by at least two different authors to ensure reproducibility. All attempts at replication were successful.
Randomization	Insects were randomly assigned to treatment groups.
Blinding	Treatment groups were assigned description-free codes (e.g., 'Treatment A') during data collection and analysis.

Reporting for specific materials, systems and methods

We require information from authors about some types of materials, experimental systems and methods used in many studies. Here, indicate whether each material, system or method listed is relevant to your study. If you are not sure if a list item applies to your research, read the appropriate section before selecting a response.

Materials & experimental systems

n/a	Involved in the study	
☒	☐	Antibodies
☒	☐	Eukaryotic cell lines
☒	☐	Palaeontology and archaeology
☐	☒	Animals and other organisms
☒	☐	Clinical data
☒	☐	Dual use research of concern
☒	☐	Plants

Methods

n/a	Involved in the study	
☒	☐	ChIP-seq
☒	☐	Flow cytometry
☒	☐	MRI-based neuroimaging

Animals and other research organisms

Policy information about studies involving animals; ARRIVE guidelines recommended for reporting animal research, and Sex and Gender in Research

Laboratory animals	A laboratory colony of Psylliodes chrysocephala was used in the study. Newly emerging adults collected from this colony were used during the study. The laboratory culture of P. chrysocephala was first established by Franziska Beran in 2012 using adults collected in Laasdorf, Thuringia, Germany.
Wild animals	The study did not involve wild animals.
Reporting on sex	The study focused on female Psylliodes chrysocephala because the aestivation is more prominent in the females compared to males in this species.
Field-collected samples	The study did not involve field-collected samples
Ethics oversight	Ethical approval or guidance was not required for this study as Psylliodes chrysocephala is an invertebrate species.

Note that full information on the approval of the study protocol must also be provided in the manuscript.

Plants

Seed stocks	*Report on the source of all seed stocks or other plant material used. If applicable, state the seed stock centre and catalogue number. If plant specimens were collected from the field, describe the collection location, date and sampling procedures.*
Novel plant genotypes	*Describe the methods by which all novel plant genotypes were produced. This includes those generated by transgenic approaches, gene editing, chemical/radiation-based mutagenesis and hybridization. For transgenic lines, describe the transformation method, the number of independent lines analyzed and the generation upon which experiments were performed. For gene-edited lines, describe the editor used, the endogenous sequence targeted for editing, the targeting guide RNA sequence (if applicable) and how the editor was applied.*
Authentication	*Describe any authentication procedures for each seed stock used or novel genotype generated. Describe any experiments used to assess the effect of a mutation and, where applicable, how potential secondary effects (e.g. second site T-DNA insertions, mosiacism, off-target gene editing) were examined.*
